# Design of a Novel MEMS Microgripper with Rotatory Electrostatic Comb-Drive Actuators for Biomedical Applications

**DOI:** 10.3390/s18051664

**Published:** 2018-05-22

**Authors:** Luis A. Velosa-Moncada, Luz Antonio Aguilera-Cortés, Max A. González-Palacios, Jean-Pierre Raskin, Agustin L. Herrera-May

**Affiliations:** 1 Departamento de Ingeniería Mecánica, DICIS, Universidad de Guanajuato/Carretera Salamanca-Valle de Santiago km 3.5+1.8, Salamanca 36885, Mexico; aguilera@ugto.mx (L.A.A.-C.); maxg@ugto.mx (M.A.G.-P.); 2 Grupo de Investigación GIDEATIC, Universidad Popular del Cesar Seccional Aguachica, Carrera 40 via al Mar, Aguachica 25010, Colombia; 3 Institute of Information and Communication Technologies, Electronics and Applied Mathematics (ICTEAM), Université catholique de Louvain (UCL), 1348 Louvain-la-Neuve, Belgium; jean-pierre.raskin@uclouvain.be; 4 Micro and Nanotechnology Research Center, Universidad Veracruzana, Calzada Ruíz Cortines 455, Boca del Río 94294, Mexico; leherrera@uv.mx; 5 Maestría en Ingeniería Aplicada, Facultad de Ingeniería de la Construcción y el Hábitat, Universidad Veracruzana, Calzada Ruíz Cortines 455, Boca del Río 94294, Mexico

**Keywords:** cancer, circulating tumor cell, comb drive actuators, FEM, MEMS, microgripper, polysilicon resonator, SUMMiT V

## Abstract

Primary tumors of patients can release circulating tumor cells (CTCs) to flow inside of their blood. The CTCs have different mechanical properties in comparison with red and white blood cells, and their detection may be employed to study the efficiency of medical treatments against cancer. We present the design of a novel MEMS microgripper with rotatory electrostatic comb-drive actuators for mechanical properties characterization of cells. The microgripper has a compact structural configuration of four polysilicon layers and a simple performance that control the opening and closing displacements of the microgripper tips. The microgripper has a mobile arm, a fixed arm, two different actuators and two serpentine springs, which are designed based on the SUMMiT V surface micromachining process from Sandia National Laboratories. The proposed microgripper operates at its first rotational resonant frequency and its mobile arm has a controlled displacement of 40 µm at both opening and closing directions using dc and ac bias voltages. Analytical models are developed to predict the stiffness, damping forces and first torsional resonant frequency of the microgripper. In addition, finite element method (FEM) models are obtained to estimate the mechanical behavior of the microgripper. The results of the analytical models agree very well respect to FEM simulations. The microgripper has a first rotational resonant frequency of 463.8 Hz without gripped cell and it can operate up to with maximum dc and ac voltages of 23.4 V and 129.2 V, respectively. Based on the results of the analytical and FEM models about the performance of the proposed microgripper, it could be used as a dispositive for mechanical properties characterization of circulating tumor cells (CTCs).

## 1. Introduction

Cancer is a major public health problem worldwide and the second leading cause of death in the world, which generated 8.8 million deaths in 2015 [1]. For instance, in the United States the cancer is second cause of human deaths, where metastasis generates 90% of all cases [2]. More researches about efficient treatments and rapid diagnostic testing of cancer are necessary to reduce the mortality related with this disease. For instance, circulating tumor cells (CTCs) inside the human blood represent the beginning of the process of blood-borne cancer metastasis [3,4]. These cells escaped from primary tumors of patients to flow inside their blood [5], whose CTCs levels is correlated with the onset of later metastatic relapse [6,7,8] or with the survival of patients with overt metastasis [9,10,11]. Devices that capture CTCs of patient blood samples could be used to detect earliest signs of tumor metastasis. Recently, several researchers [12,13,14,15,16,17] have developed microfluidic platforms to separate CTCs of a blood sample considering that these cells have larger size respect to red and white blood cells. Thus, microfluidic platforms isolate the largest cells that could be CTCs considering these cells with dimensions larger than 20 μm [18]. Meng et al. [19] measured the mean dimension and standard deviation of CTCs in patient blood samples with breast cancer primary tumor from 32.0 μm to 5.8 μm, respectively. However, Moreno et al. [20] reported CTCs with size larger than 10 μm in patient blood samples with metastatic carcinoma of prostate. However, typical sizes of blood cells are 5 μm to 9 μm for erythrocytes, 10 μm to 15 μm for granulocytes, 7 μm to 18 μm for lymphocytes and 12 μm to 20 μm for monocytes [21,22,23]. Due to these variations in the dimensions of the cells is necessary to design novel devices to guarantee a successful identification of CTCs from red and white blood samples. For this reason, some researchers [24,25,26] have studied mechanical properties of CTCs and their relation with the metastasis. In particular, stiffness variation of the cancer cells is related with the cancer progression [27]. Some techniques have been used to study the stiffness of cancer cells such as atomic force microscope (AFM) [28,29,30,31,32], optical lasers [33,34] and micropipette aspiration [35]. These studies have reported that cancer cells are generally softer, more deformable and more contractible than non-malignant cells [36]. These stiffness values of the cells are related with the magnitude of their elastic modulus. Thus, the detection of stiffness or elastic modulus of cells could be used to identify CTCs and study the cancer progression in patients as well as the efficiency of medical treatments against cancer. In addition, the evaluation of these mechanical parameters of cells could be employed for detecting the early stage development of cancer.

Microelectromechanical systems (MEMS) have allowed the development of microgrippers, which could be used for manipulation and mechanical characterization of cells. These microgrippers will measure the mechanical properties of soft materials as cells or biological tissues. These mechanisms have small size, high precision, wide operation range and low power consumption; in addition, their displacements could be controlled to manipulate cells [37]. Several MEMS microgrippers have been designed for mechanical testing of different materials at the microscale [38,39,40]. Generally, the motion of microgrippers is reached with electrothermal or electrostatic actuators. For instance, Qu et al. [38] developed a microgripper with two electrothermal actuators and capacitive force sensors for mechanical characterization of soft materials. This microgripper has mobile parts of high flexibility, which permit real-time control of gripping strength. However, several of the MEMS microgrippers have limited motion and their actuation mechanisms need large area to increase the driving force. This problem can be solved using MEMS microgrippers with rotatory comb-drive actuators [40,41], which may rotate respect to a pivot [42,43]. In general, the manipulation and characterization of biological cells demands microgrippers with real-time control system and high resolution. In order to characterize the mechanical properties of CTCs, we designed a novel MEMS microgripper with rotatory comb-drive actuators with advantages such as small size, simple operation principle, low actuation voltage and wide displacements range. This microgripper design is based on the SUMMiT V fabrication process from Sandia National Laboratories, allowing three mobile polysilicon structural layers that increase the numbers of electrodes without expanding of the working area. This design enables a controlled motion in both opening-closing directions of the rotatory comb-drive actuator.

This paper is organized as follows. Section 2 includes the design and modeling of the microgripper mechanical performance. Section 3 describes the results and discussions of the mechanical behavior of the microgripper. Finally, Section 4 contains the conclusions and future works.

## 2. Design and Modeling

This section reports the design and modeling of the microgripper mechanical behavior, considering analytical and finite element method (FEM) models.

### 2.1. Microgripper Design

**Figure 1:**
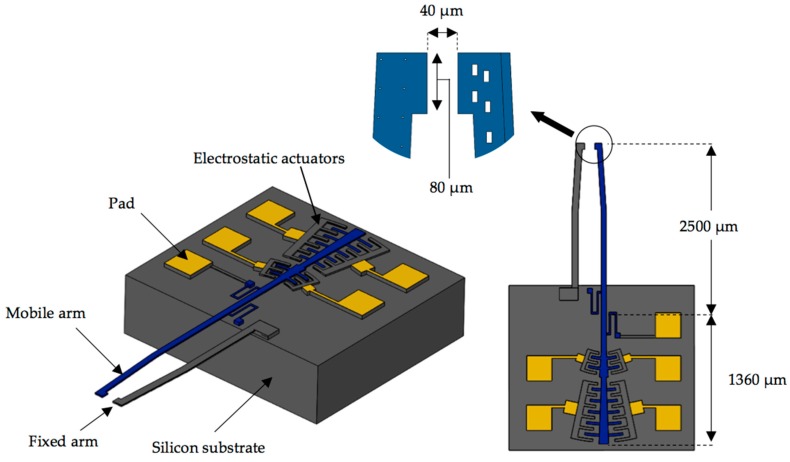
Schematic view of the MEMS microgripper design based on the SUMMiT V fabrication process form Sandia National Laboratories.

**Figure 2:**
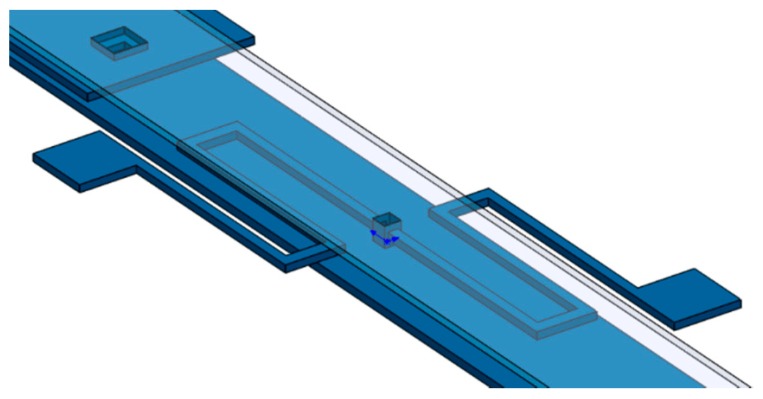
3D view of the serpentine springs that support the mobile arm of the microgripper.

Figure 1 depicts the schematic view of the microgripper design SUMMiT V surface-micromachining process from Sandia National Laboratories [44]. This design has rotatory comb-drive actuators and two polysilicon arms (one fixed and one mobile), which the tips of these arms will be used to squeeze a cell. A first polysilicon arm is clamped on the silicon substrate and second arm is supported by two polysilicon serpentine springs, as shown in Figure 2. The junction point of these springs with the mobile arm is located on the mass center of mobile arm. This second arm has rotational motion due to electrodynamic and electrostatic actuators, which allows to open and close the tip of the mobile arm of the microgripper. The initial open of the tips of the microgripper is 40 μm, which is a distance larger than the cell size. Due to the rotational motion of the mobile arm, this initial distance can be increased or decreased. On the other hand, the electrodynamic and electrostatic com-drive actuators can be supplied by ac and dc voltages, respectively. These actuators will allow the rotational motion of the mobile arm through electrostatic forces. With this actuators design, we can decrease the surface area of the microgripper.

**Figure 3:**
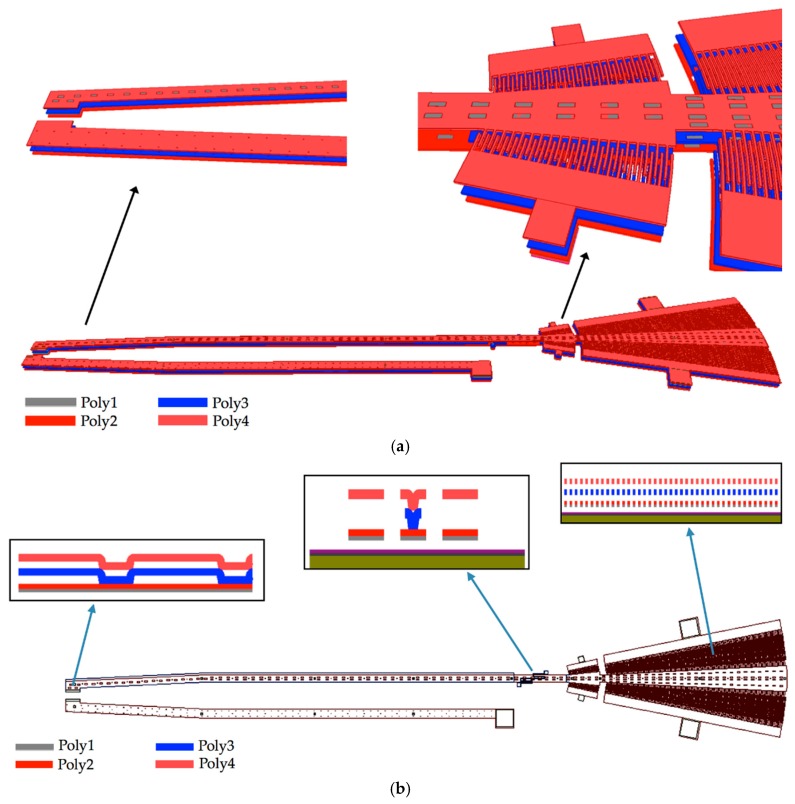
(**a**) 3D view and (**b**) layout of the microgripper based on the SUMMiT V surface-micromachining process.

The microgripper die will have a post-processing to etch a section of the silicon substrate, silicon dioxide and nitride layers below of the microgripper arms. Sacrificial oxide layers will protect the microgripper arms and actuators during the post-processing. Finally, the sacrificial oxide layers will be etched to release the microgripper arms and actuators. The structural design of the microgripper considers three mobile structural layers (see Figure 3) to increase the number of electrodes and electrostatic torque. The first mobile structural layer is composed by the attaching of poly1 and poly2 layers of the SUMMiT V process. The mobile second and third polysilicon layers are integrated by poly3 and poly4 layers of the SUMMiT V process, respectively. In each polysilion layer, the mobile arm contains at its end 120 pairs of comb-drive electrodes (electrostatic actuators) that are supplied with dc voltage. In addition, the mobile arm has 20 pairs of comb-drive electrodes in each polysilicon layer, forming the electrodynamic actuators that are supplied with dc and ac voltages. With both electrodes and under an actuation voltage, the mobile arm can open and close the microgripper tips, achieving a maximum opening displacement of 80 µm. This opening displacement between the microgripper tips can be controlled adjusting the actuation voltages. The rotatory electrodes at one side of the mobile arm are used as actuation electrodes and those located at the other side are employed as sensing electrodes. The sensing electrodes detect capacitance shifts that are related with the angular motion of the mobile arm. These electrodes are used to real-time adjust the actuation voltage and to control the displacements of the mobile arm. Thus, the opening and closing displacements of the microgripper can be controlled adjusting the actuation voltages. The electrostatic and electrodynamic actuators have independent electronic circuits, which allow their performance under different voltages without instability problems [45]. The electrostatic actuators are used to open and close the microgripper before catching the cell. After gripping the cell, electrodynamic actuators are employed to squeeze the cell. The serpentine springs (see Figure 3) support the mobile arm using a simple polysilicon beams array. The mobile arm has a rotational motion around the connection point with the serpentine springs for different values of actuation voltages.

**Figure 4:**
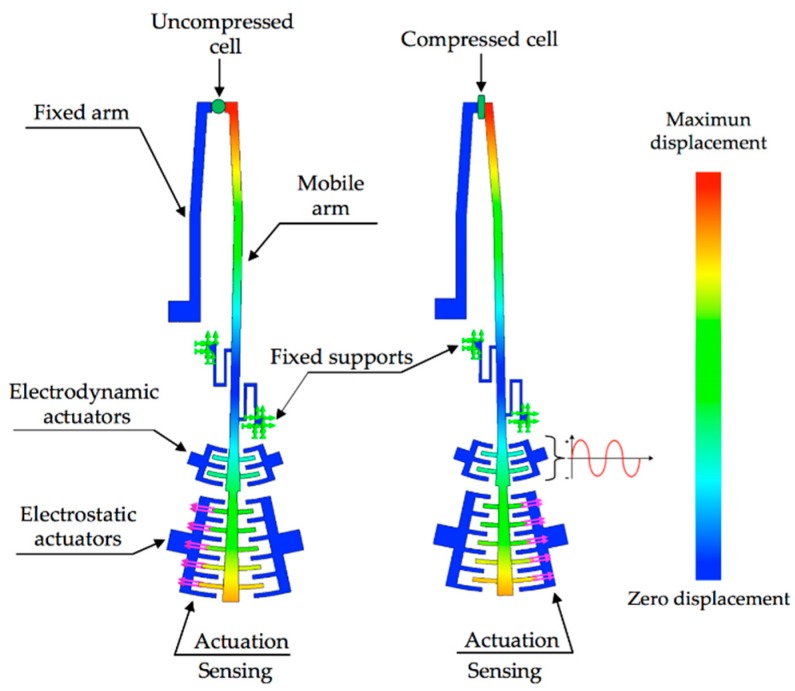
Schematic diagram of the MEMS microgripper operation with rotatory comb-drive actuators.

Figure 4 depicts the operation principle of the microgripper for monitoring stiffness and elastic modulus of CTCs. Adjustable actuation voltages are applied to comb-drive electrodes that generate electrostatic torques at the mobile arm, which allows opening and closing displacements between the microgripper tips. These displacements can be adjusted to catch potential CTCs after that they are filtered from a patient blood sample using a microfluidic platform [12,13,14,15,16,17]. First a dc bias voltage will be applied at the electrostatic comb drive actuators to produce an electrostatic torque that moves the mobile arm at the closing direction of microgripper. Next, the dc bias voltage is modified to adjust the closing displacement of the microgripper and thus it can capture one cell. Then, an ac bias voltage is supplied at the electrodynamic comb drive actuators to produce small alternating electrostatic forces that generate oscillating motions of the mobile arm. Thus, the microgripper tips squeeze the cell with an alternating electrostatic force and the displacements of the deformed cell can be measured through the capacitance shift of the electrostatic actuators. These variations of the displacements and capacitances are related with the elastic modulus and stiffness of the gripped cell. Finally, the measurements of these mechanical parameters of the gripped cell will be compared with the elastic modulus and stiffness values of CTCs from different tumor types. If the measured elastic modulus of the compressed cell matches with that of CTCs then the gripped cell originates from a tumor.

An option to measure the stiffness of CTCs can be obtained increasing the actuation frequency of the ac voltage up to reach the torsional resonant frequency of the mobile arm. The stiffness of the cell increases the self-stiffness of the mobile arm changing its resonant frequency. This frequency shift will be related with the stiffness of the gripped cell and compared with the stiffness values of CTCs.

### 2.2. Modeling of the Microgripper Performance

The elastic modulus or stiffness of gripped cells are extracted from the variations of the capacitances or rotational resonant frequencies of the microgripper. The total capacitance *C* of the electrodes for the electrostatic actuators is obtained as [40,41,46,47]:

(1) $$C=\varepsilon_{0}\theta h\left[{\sum_{i=1}^{n-1}\left(\ln\frac{R_{0}+2i\left(W_{f}+g\right)}{R_{0}+2i\left(W_{f}+g\right)-g}\right)}^{-1}+{\sum_{i=1}^{n-1}\left(\ln\frac{R_{0}+\left(2i+1\right)\left(W_{f}+g\right)}{R_{0}+2i\left(W_{f}+g\right)+W_{f}}\right)}^{-1}\right].$$

The actuation torque (*τ*) on the mobile arm of the microgripper can be determined by [46,47,48,49]:

(2) $$\tau =\frac{1}{2}\left(\frac{\partial C}{\partial \theta }\right)V^{2},$$

(3) $$\tau =\frac{1}{2}\varepsilon_{0}h\left[{\sum_{i=1}^{n-1}\left(\ln\frac{R_{0}+2i\left(W_{f}+g\right)}{R_{0}+2i\left(W_{f}+g\right)-g}\right)}^{-1}+{\sum_{i=1}^{n-1}\left(\ln\frac{R_{0}+\left(2i+1\right)\left(W_{f}+g\right)}{R_{0}+2i\left(W_{f}+g\right)+W_{f}}\right)}^{-1}\right]V^{2}=\tau_{0}V^{2},$$

where *ε*_0_ is the air permittivity, *θ* is the overlap angle between the comb drive electrodes, *h* is the thickness of the comb drive electrodes, *R_0_* is the inner radius of the first comb drive electrode close to the pivot point, *W_f_* is the width of the comb drive electrodes, *g* is the gap distance between the comb drive electrodes, *n* is the number of comb drive electrodes and *V* is the actuation voltage.

**Figure 5:**
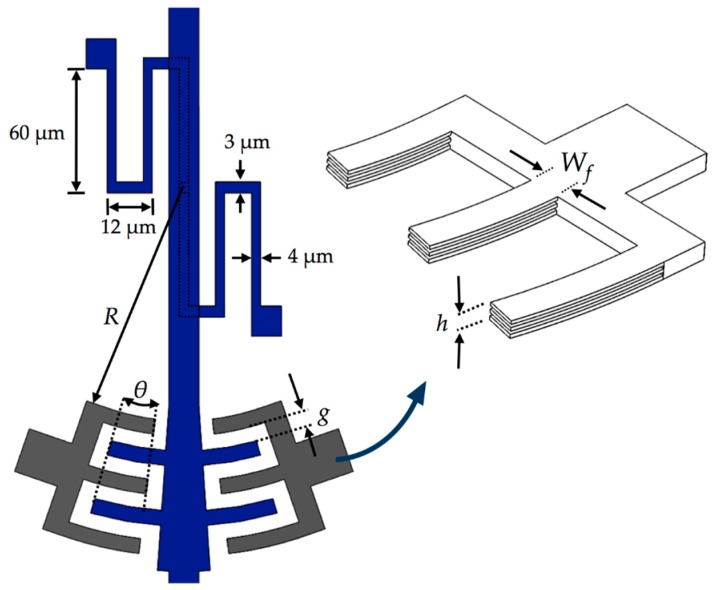
Schematic view of the electrodynamic actuators which are used to measure the capacitance shifts of the designed microgripper.

Figure 5 depicts a schematic view of the electrodynamic actuators of the microgripper used to measure the capacitance variations. Table 1 indicates the dimensions of the electrodynamic electrodes.

The dimensions of these actuators were selected to decrease stiffness of the serpentine springs, considering on the design rules of the SUMMiT V fabrication process. For instance, the gap (*g*) between comb drive electrodes has a minimum distance of 2 μm that allows the design rules of the SUMMiT V process. This distance is suitable to increase the actuation force between the electrodes of the electrodynamic actuators.

#### 2.2.1. Modeling of the Mobile Arm

The mobile arm of the microgripper has rotational motion around a pivot point between the serpentine springs, in where the arm has its mass center. The rotational displacement of the mobile arm can be estimated using Newton-Euler equation around the *z*-axis in the pivot point:

(4) $$\sum_{}^{}T=J_{b}\alpha ,$$

where *T* represents the torsional moments acting on the mobile arm, *J_b_* is the polar moment of inertia of the mobile arm and *α* is the angular acceleration of the mobile arm.

**Figure 6:**
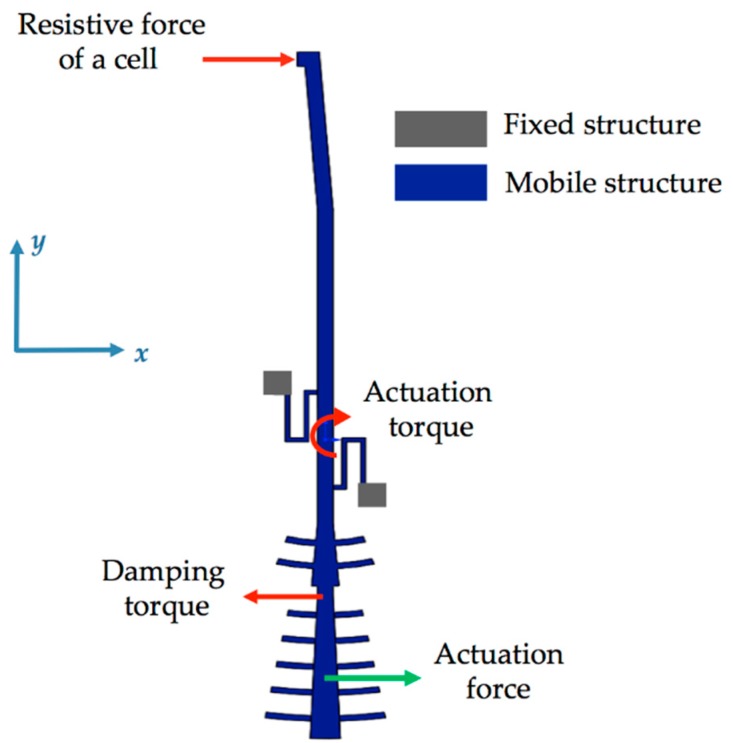
Free body diagram of the forces and torsional moments on the mobile arm of the microgripper.

Figure 6 shows the torsional moments generated by the electrostatic actuation, the damping and resistive forces on the mobile arm of the microgripper.

Considering all torsional moments on mobile arm and substituting them in Equation (4), we obtain the following equation:

(5) $$T_{a}-T_{d}-T_{s}-T_{c}=J_{b}\alpha ,$$

where *T_a_*, *T_d_*, *T_s_* and *T_c_* are the actuation and damping torques, reaction torque of the serpentine springs and torque generated by the resistive force of a gripped cell, respectively. 

For electrostatic actuation, the motion equation of the mobile arm is reduced as:

(6) $$T_{a}-T_{s}-T_{c}=0.$$

#### 2.2.2. Modeling of the Electrostatic Actuation Torque

**Figure 7:**
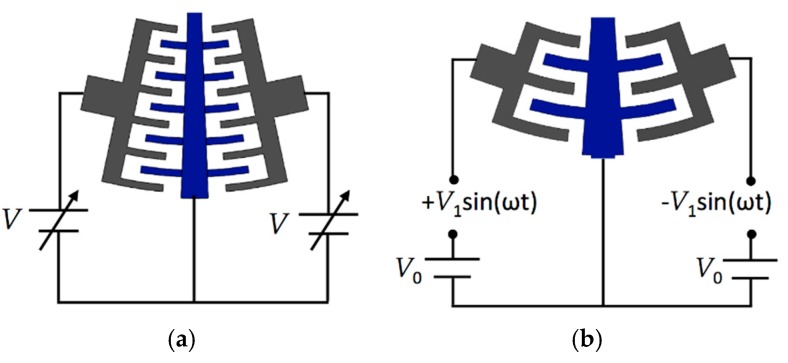
Schematic view of the electrical circuits with the dc and ac bias voltages of (**a**) electrostatic and (**b**) electrodynamic actuators of the microgripper.

The interdigitated comb drive capacitors can be used for actuation or sensing. Figure 6 shows the electronic circuits for the electrostatic and electrodynamic actuators of the microgripper. For the electrostatic actuation, dc bias voltage (*V*) is applied to each side of the comb drive electrodes to achieve rotational motion of the mobile arm (see Figure 7a). On the other hand, the electrodynamic actuation is obtained providing an alternating voltage through a push-pull driving (see Figure 7b). A push-pull driving circuit is often considered as the best solution for driving when the forces are applied to the mobile structure from both sides, as in the case of the microgripper. For the electrodynamic actuators, ac voltages (*V_L_* and *V_R_*) supplied to the left and right sides of the push-pull driving are given as [45]:

(7) $$V_{L}=V_{0}+V_{1}\sin(\omega t),$$

(8) $$V_{R}=V_{0}-V_{1}\sin(\omega t).$$

The resulting driving voltage on the comb drive actuator is [45]:

(9) $$V_{L}^{2}-V_{R}^{2}=4V_{0}V_{1}\sin(\omega t).$$

Thus, the alternative actuation torque can be rewritten as

(10) $$T_{a}=\tau_{0}V^{2}=4V_{0}V_{1}\sin(\omega t)=B\sin(\omega t).$$

In order to avoid the instability of the actuators, the maximum voltage (*V_c_*) supplied to the actuators is given by [45]:

(11) $$V_{c}=\sqrt{\frac{kg^{2}}{C}},$$

where *k* is the linear stiffness along the normal direction of the comb drive electrodes, *g* is the gap distance between the comb drive electrodes (see Figure 4) and *C* is the total capacitance defined by Equation (1).

#### 2.2.3. Modeling of the Damping Torque

**Figure 8:**
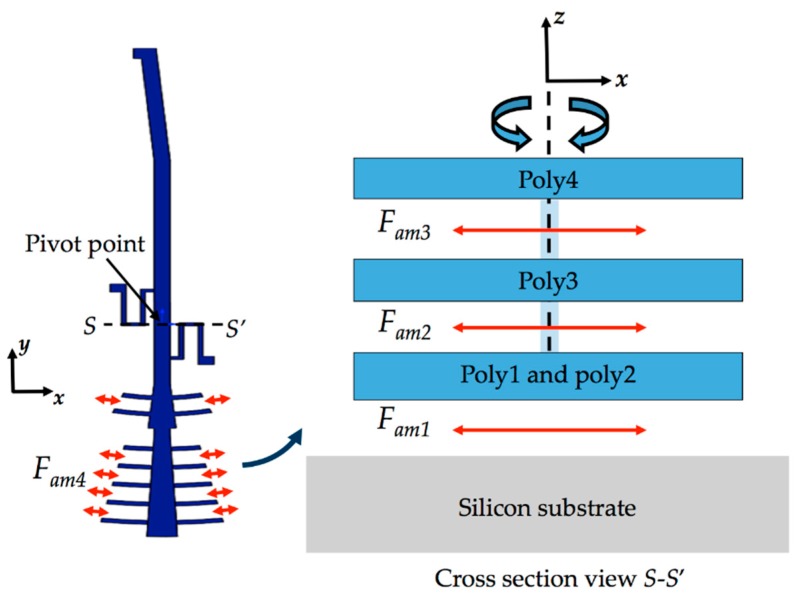
Schematic diagram of the air damping forces caused by rotational motion of the mobile arm of the microgripper.

**Figure 9:**
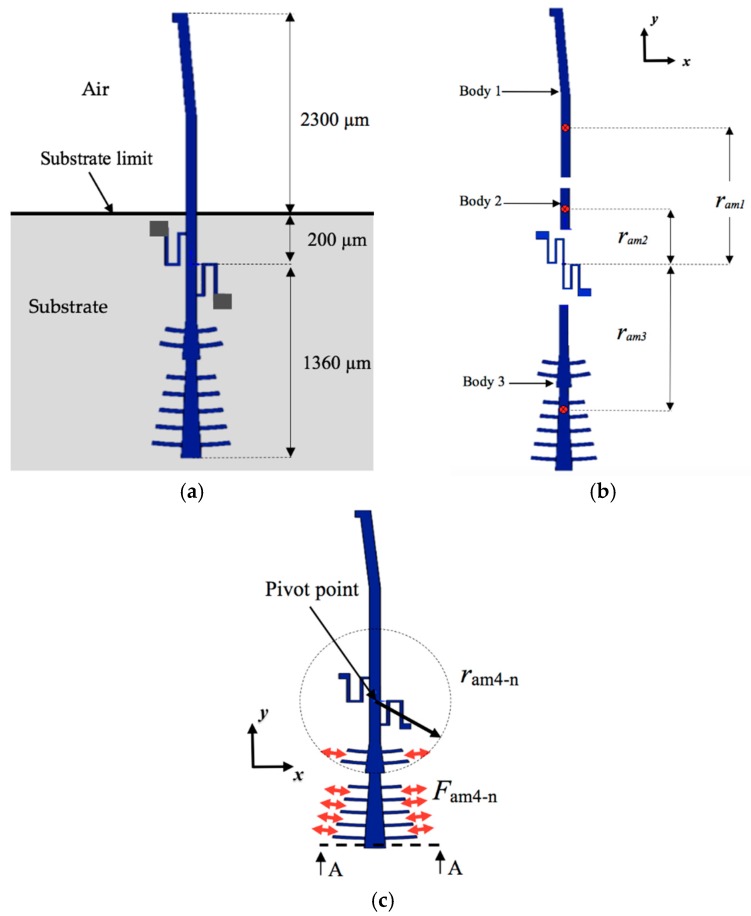
Schematic diagram of the mobile arm considered to calculate the air damping forces: (**a**) dimensions of the three main sections; (**b**) distances of the three first damping force vectors (*F_am_*_1_, *F_am_*_2_ and *F_am_*_3_) respect to the pivot point; and (**c**) distance of the fourth damping force (*F_am_*_4_) respect to the pivot point.

The design of the microgripper allows that only its tips can have contact with the CTCs, which enable that the most of the mobile structural layers oscillate around the air environment. This interaction between the movable structural layers and air generates energy dissipation (i.e., air damping). Figure 8 depicts the air damping forces generated by the rotational displacements of the microgripper keeping a constant gap respect to substrate. These air-damping forces can be expressed as function of angular velocity ${\dot{\theta }}_{z}$ of the mobile arm and viscosity coefficient (*μ*) of the air [45]. To evaluate these air-damping forces, we consider that one part of the mobile arm of the microgripper is located above from silicon substrate, as shown in Figure 9a. Based on this assumption, the mobile arm is divided in three bodies, which only the second and third bodies are above from substrate (see Figure 9b). Thus, air-damping forces can be expressed as function of angular velocity of the mobile arm and the viscosity coefficient (*μ*) of the air. For the first mobile layer (composed by the junction between poly1 and poly2 films) of the second and third section (bodies 2 and 3) of the microgripper, the slide-film air damping force (*F_am_*_1_) is obtained by

(12) $$F_{am1}=F_{am1-b2}+F_{am1-b3}=\mu \frac{A_{p-b2}}{d_{p}}r_{am-b2}^{2}{\dot{\theta }}_{z}+\mu \frac{A_{p-b3}}{d_{p}}r_{am-b3}^{2}{\dot{\theta }}_{z}=c_{am1}{\dot{\theta }}_{z},$$

where *F_am_*_1*-b*2_ and *F_am_*_1*-b*3_ are the slide-film air damping forces in bodies 2 and 3 of the mobile arm, respectively, *A_p-b_*_2_ and *A_p-b_*_3_ are the effective surface area of both bodies 2 and 3, and *d_p_* is the gap between the first mobile layer and the silicon substrate.

The slide-film air damping forces (*F_am_*_2_ and *F_am_*_3_) due to the mobile second (poly3) and third (poly4) layers of the microgripper are determined by

(13) $$F_{am2}=F_{am2-b1}+F_{am2-b2}+F_{am2-b3}=c_{am2}{\dot{\theta }}_{z},$$

with

(14) $$F_{am2-b1}=\frac{32}{3}\mu l_{am1}r_{am1}^{2}{\dot{\theta }}_{z},$$

(15) $$F_{am2-b2}=\frac{32}{3}\mu l_{am2}r_{am2}^{2}{\dot{\theta }}_{z},$$

(16) $$F_{am2-b3}=\frac{32}{3}\mu l_{am3}r_{am3}^{2}{\dot{\theta }}_{z},$$

(17) $$F_{am3}=F_{am2},$$

where *l_am_*_1_, *l_am_*_2_ and *l_am_*_3_ are the length half of bodies 1, 2 and 3, respectively, *r_am_*_1_, *r_am_*_2_ and *r_am_*_3_ are the distances, parallel to plane *xy*, between the damping force vectors of bodies 1, 2 and 3, respectively.

The slide-film air damping force (*F_am_*_4_) on the sidewalls of the mobile comb drive electrodes of the actuators can be expressed as:

(18) $$F_{am4}=\sum_{0}^{n-1}\frac{\mu A_{sn}}{g}r_{am4-n}^{2}{\dot{\theta }}_{z}=c_{am4}{\dot{\theta }}_{z},$$

where *A_sn_* is the area of sidewalls of each mobile comb-drive electrode, *n* is the number of mobile electrodes, *g* is the gap between the sidewalls and fixed comb drive electrodes and *r_am_*_4*-n*_ is the distance between each mobile comb and the pivot point (see Figure 8c).

The total damping force (*F_d_*) is calculated by

(19) $$F_{d}=F_{am1}+F_{am2}+F_{am3}+F_{am4}=c_{amT}{\dot{\theta }}_{z}$$

(20) $$c_{amT}=c_{am1}+2c_{am2}+c_{am4}.$$

#### 2.2.4. Modeling of the Reaction Torque of the Serpentine Springs

**Figure 10:**
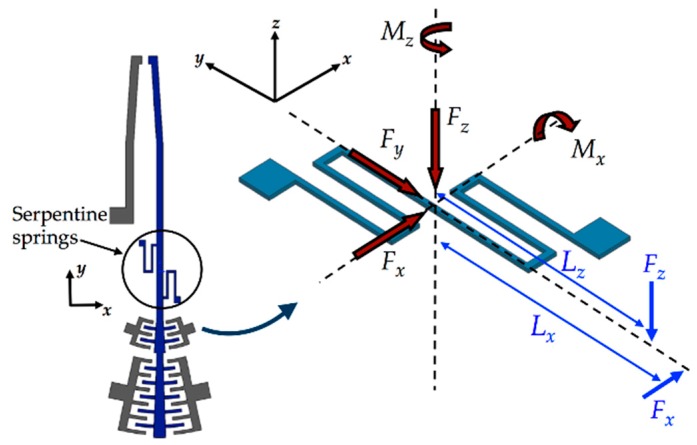
Schematic diagram of the forces applied on the serpentine springs of the mobile arm of the microgripper.

Displacements of the mobile arm depend on the electrostatic actuation forces, damping forces and stiffness of the serpentine springs. Figure 10 illustrates all the forces and moments on the serpentine springs. *F_x_*, *F_y_* and *F_z_* represent the actuation force and opposition forces caused by substrates and weight of the mobile arm, respectively, and *M_x_* and *M_z_* represent the moments produced by the forces *F_z_* and *F_x_*, respectively.

Serpentine springs of the mobile arm are very flexible when are subjected to torsional moment *M_z_*, which allows the arm rotation around *z*-axis direction. Considering small rotation of the mobile arm around *z*-axis direction and constant cross section of the serpentine springs, the angular displacement *θ_zMz_* generated by *M_z_* is determined through Castigliano’s second theorem [48]:

(21) $$\theta_{zMz}=\frac{\partial U}{\partial M_{z}},$$

where *U* is the total strain energy of the serpentine springs.

The total strain energy of the serpentine springs includes strain energies due to the tension or compression force as well as torsional and bending moments, which are obtained by [48]:

(22) $$U=U_{tension}+U_{torsion}+U_{bending},$$

(23) $$U_{tension}=\int_{L}\frac{F^{2}}{2EA}dx,$$

(24) $$U_{torsion}=\int_{L}\frac{\tau^{2}}{2GJ}dx,$$

(25) $$U_{bending}=\int_{L}\frac{M^{2}}{2EI}dx,$$

where *F*, *τ* and *M* are the tension or compression force, torsional and bending moments, respectively, *L* and *A* are the length and cross-section area of each beam that composes the serpentine springs, respectively, *E* and *G* are the elastic and shear modulus of the polysilicon, respectively, *J* and *I* are the polar moment of inertia and moment of inertia of the serpentine spring.

The moment of inertia and polar moment of inertia of the rectangular cross-section of the serpentine spring are calculated as [49]:

(26) $$I=\frac{bh}{12},$$

(27) $$J=\frac{bh^{3}}{3}\left(1-\frac{192h}{\pi^{5}b}\sum_{m=0}^{\infty }\frac{1}{{\left(2m+1\right)}^{5}}\tanh\left[\frac{(2m+1)\pi b}{2h}\right]\right),$$

where *b* and *h* are the width and height of the beam of the serpentine springs, respectively. 

The rotational stiffness around *z*-axis direction of the serpentine spring (*k_tz_*) is given by:

(28) $$k_{tz}=\frac{M_{z}}{\theta_{zMz}}$$

The reaction torque of the serpentine spring is approximated by [50]:

(29) $$T_{s}=k_{tz}\theta_{z}$$

By applying the Castigliano’s second theorem, we determine the following torsional and bending stiffness of the serpentine spring: *k_tz_* = 49,557 μN μm rad^−1^, *k_tx_* = 11,982 μN μm rad^−1^, *k_Fx_* = 25.74 μN μm^−1^, *k_Fy_* = 90.76 μN μm^−1^ and *k_Fz_* = 10.14 μN μm^−1^.

#### 2.2.5. Modeling of the Resistive Torque of the Cell

**Figure 11:**
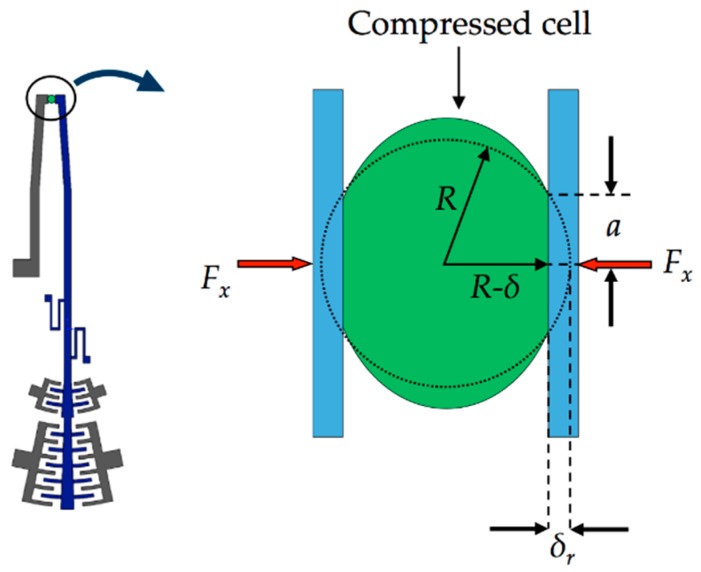
Schematic view of the Hertzian mechanics model for large deformations used to estimate the resistive force of a cell.

The stiffness of the mobile arm is altered when it has contact with the cell, which offers an opposite resistive force to the compression actuation force of the mobile arm. Therefore, an increase of this force is necessary to squeeze the cell. The magnitude of resistive force can be estimated using the Hertzian mechanics model for large deformations (see Figure 11). This model calculates the mathematical relationship between the compression force and normal displacement of the microgripper tip considering nonlinear elasticity and lateral extension of the compressed cell. We assumed that the microgripper tips apply compression force along the normal direction of the cell. 

The mathematical relationship between displacement and compression force can be estimated by [51,52]:

(30) $$F_{xc}=\frac{\delta_{r}E_{c}}{\frac{3(1-v^{2})}{4a}-\frac{f(a)}{\pi }},$$

(31) $$f(a)=\frac{2(1+v)R^{2}}{{\left(a^{2}+4R^{2}\right)}^{\frac{3}{2}}}+\frac{1-v^{2}}{{\left(a^{2}+4R^{2}\right)}^{\frac{1}{2}}},$$

where *F_xc_* is the compression force that deforms the cell, *R* is the cell radius, *δ_r_* is the displacement of the cell along of its radial direction, *a* is the radius of contact area of the cell, and *E_c_* and *v* are the elastic modulus and Poisson ratio of the compressed cell, respectively. 

We used the values of elastic modulus and Poisson ratio of the cell reported in [53]. The radius of the contact area of the gripped cell is estimated by

(32) $$a=\sqrt{2R\delta_{r}-\delta_{r}{}^{2}}$$

Resistive torque (*T_c_*) caused by the compression force of the cell respect to the pivot point is approximated by:

(33) $$T_{c}=F_{xc}r_{c},$$

where *r_c_* is the distance of the compression force of the cell to the pivot point and *r_c_* = 2*δ_r_*/*θ_z_*.

### 2.3. Modeling of the Resonant Frequency

The value of the first rotational resonant frequency of the mobile arm changes due to the additional stiffness that provides the gripped cell. This resonant frequency shift will be used to estimate the elasticity of the gripped cells. We use a lumped parameter model to predict the variation of resonant frequency of the rotational vibration mode on the plane *xy* (i.e., around *z*-axis) of the mobile arm. For this, the mobile arm is assumed with an equivalent mass moment of inertia (*J_b_*) and a torsional spring of equivalent stiffness (*k_zt_*), both concentrated at the mass center of mobile arm. Thus, the first resonant frequency of the rotational mode around *z*-axis of the mobile arm is given by [54]:

(34) $$f_{rz}=\frac{1}{2\pi }\sqrt{\frac{k_{Tz}}{J_{b}}},$$

where *J_b_* is the polar moment of inertia of the mobile arm that is calculated around of its mass center, *k_Tz_* is the total rotational stiffness around *z*-axis direction that includes the stiffness of the serpentine springs (*k_tz_*) and cell (*k_zc_*).

(35) $$k_{Tz}=k_{tz}+k_{zc}.$$

By considering *J_b_* = 5.86 × 10^−3^ kg μm^2^, the first rotational resonant frequency of the mobile arm before of gripping the cell is 463.8 Hz.

## 3. Results and Discussions

In this section, we present the results and discussions about mechanical behavior of the microgripper based on analytical and FEM models.

**Figure 12:**
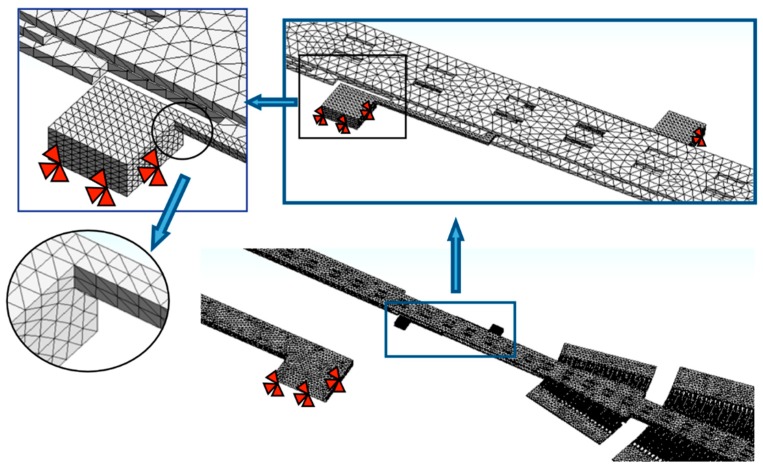
FEM model of the microgripper using solid187 elements of ANSYS software.

The stiffness modeling of the serpentine springs of the microgripper is obtained through FEM models. These models include a mesh with solid187 elements, as shown in Figure 12. The serpentine springs have refined mesh and are regarded fixed (red arrows in Figure 12) at their base, which are the joint surface with the substrate. The mechanical properties of polysilicon employed in FEM models are the follows: Young modulus of 160 GPa, Poisson ratio of 0.23 and density of 2330 kg m^−3^.

**Figure 13:**
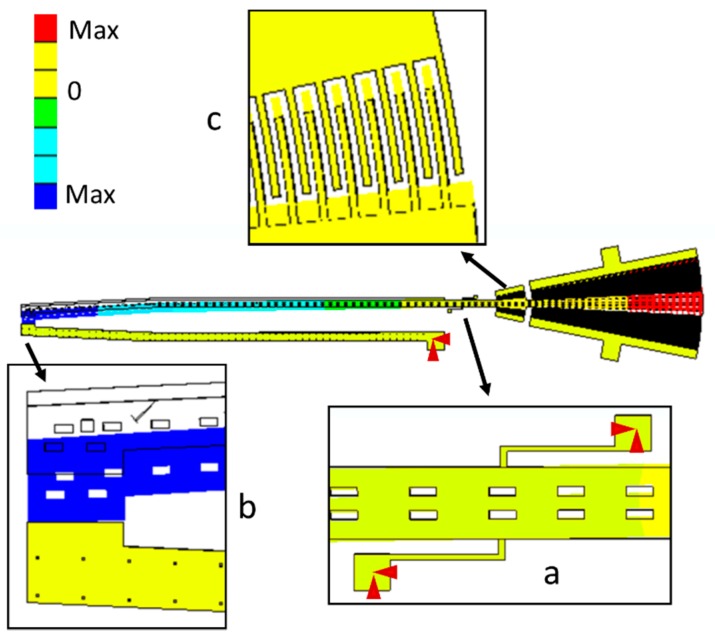
Displacements of the FEM model of the microgripper using solid187 elements of ANSYS software.

**Figure 14:**
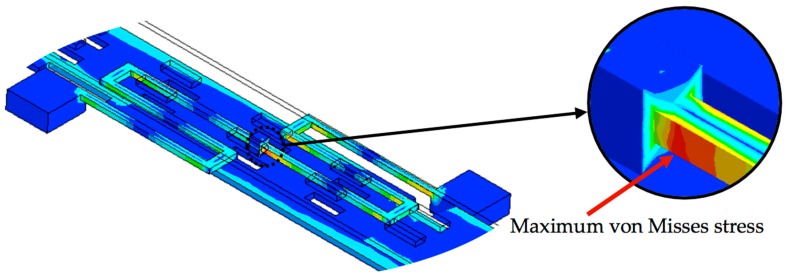
Maximum von Misses stress on the serpentine springs of the microgripper.

Figure 13 depicts the displacement of different sections of the microgripper caused by an actuation force that generates a torsional moment around *z*-axis direction (*M_z_*). In this figure, the initial position of the microgripper is represented with black lines and the final position is indicated with color lines. The mobile arm has a rotational displacement respect to its connection point with the serpentine springs. Figure 14 shows the maximum von Misses stress (111 MPa) of the microgripper located on the connection point of the serpentine springs. This value is less than the rupture stress (1 GPa) of the polysilicon, which is suitable for a safe operation of the microgripper.

**Figure 15:**
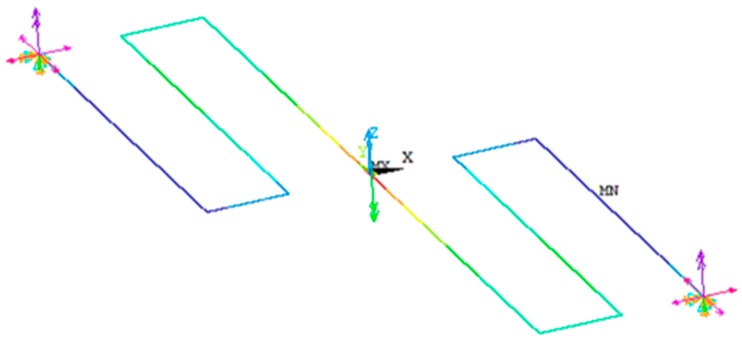
FEM model of the serpentine springs using beam188 elements of ANSYS software.

In addition, other FEM model of the serpentine springs is developed using beam188 elements, as shown in Figure 15. External points of the FEM model are considered clamped and the forces are applied on the connection point. Table 2 summarizes the stiffness results of the serpentine springs of the microgripper using the analytical and FEM models.

A modal analysis of the FEM model of the microgripper is made to predict its first resonant frequencies and vibration modes. Table 3 depicts the first five vibration modes and resonant frequencies obtained by FEM simulation of the microgripper. The rotational vibration mode around *z*-axis direction has a resonant frequency of 463 Hz. This mode is suitable for the microgripper and has a relative difference of −0.2% respect to that obtained with the analytical model.

Motion equation of the mobile arm can be rewritten as function of *θ_z_* with different operating frequencies of the ac bias voltages of the electrodynamic actuators:

(36) $$J_{b}{\ddot{\theta }}_{z}+c_{amT}{\dot{\theta }}_{z}+k_{Tz}\theta_{z}+\frac{F_{xc}r_{c}^{2}\theta_{z}}{2\delta_{r}}=B\sin(\omega t),$$

where resistive force of the cell (*F_xc_*) depends on its mechanical properties and geometry as well as the rotation angle *θ_z_*. 

To solve the differential Equation (32), the resistive torque (*T_c_*) of a cell can be obtained observing the results of the graph *T_c_ versus θ_z_*. Data of the radius (*R*) and elastic modulus (*E_c_*) of the cell are taken from experimental results of different cells types (see Table 4).

**Figure 16:**
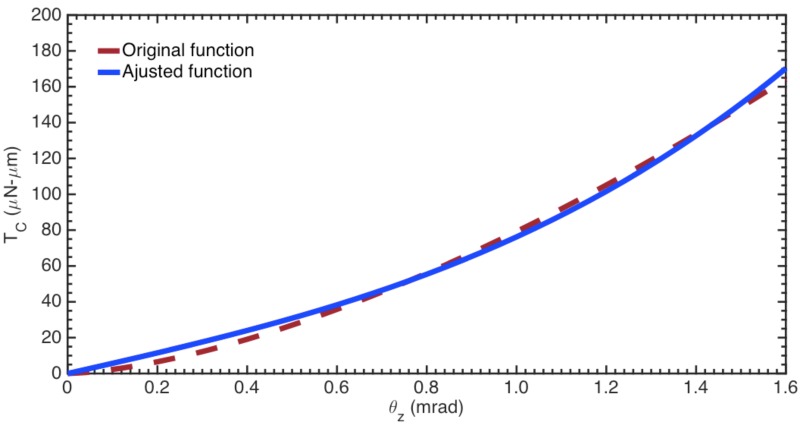
Adjusted function of the resistive torque *T_c_* for a BPH cell.

Figure 16 shows the plot *T_c_ versus θ_z_* of benign prostate hyperplasia (BHP) cells. The values of *θ_z_* are limited to 0.0016 rad due to rotation angle of the mobile arm when the cell is compressed by 20% of its radius. The results of the resistive torque of the cell have a nonlinear behavior, which can be adjusted using a third-degree polynomic function *α_a_x*^3^ + *α_b_* by applying least square method. Table 5 reports the parameters of the approximated functions (*T_c_*) for each cell type.

The motion differential equation of the mobile arm when it interacts with a biological material can be estimated by:

(37) $$J_{b}{\ddot{\theta }}_{z}+c_{amT}{\dot{\theta }}_{z}+k_{Tz}\theta_{z}+\alpha_{a}\theta_{z}+\alpha_{b}\theta_{z}^{3}=B\sin(\omega t)$$

This differential equation is known as “Duffing Equation” and it has not an exact solution; however, an approximate solution can be obtained for steady state by using the Ritz averaging method [55]. The steady-state forced vibration will involve a phase angle Ψ, whose approximate solution is given by:

(38) $$\theta_{z}=c_{1}\cos(\omega t-\Psi )=a_{1}\cos(\omega t)+b_{1}\sin(\omega t),$$

(39) $$\Psi =\tan^{-1}\left(\frac{c_{amT}\omega }{-w^{2}+k_{Tz}+\alpha_{a}+\frac{3}{4}\alpha_{b}c_{1}^{2}}\right),$$

(40) $$\frac{3\alpha_{b}c_{1}^{3}}{4(k_{Tz}+\alpha_{a})}=\left(\frac{\omega^{2}}{{\left(k_{Tz}+\alpha_{a}\right)}^{2}}-1\right)c_{1}+\frac{B}{{\left(k_{Tz}+\alpha_{a}\right)}^{2}}\sqrt{1-\frac{{\left(c_{amT}\omega c_{1}\right)}^{2}}{B^{2}}},$$

where *ω* is the angular frequency of the actuation force.

**Figure 17:**
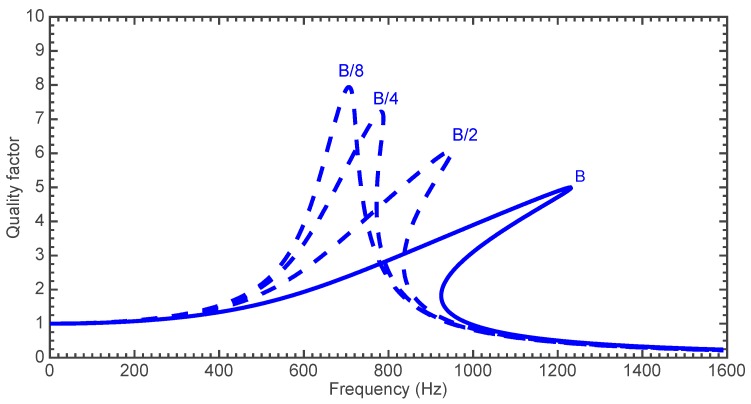
Frequency response of the microgripper when it squeezes a BHP cell.

Figure 17 shows the amplitude of the response *c*_1_ in terms of the quality factor of the microgripper, which is the number of times that its amplitude increases as function the actuation angular frequency *ω*. However, this nonlinear effect can be partially reduced when the amplitude of the actuation torque (*A*) is decreased. Figure 17 depicts the frequency response of the mobile arm when it compresses each one of three different cells types and when it operates without gripped cell.

**Figure 18:**
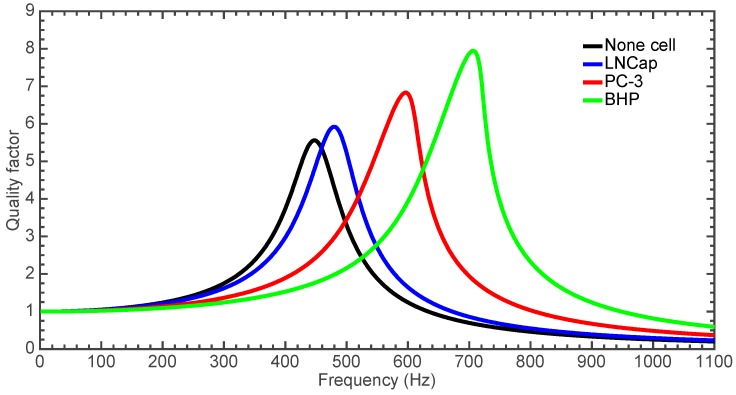
Frequency response of the microgripper when it squeezes three different cells types and without gripped cells.

Based on the results shown in Figure 18, the dynamic behavior of the microgripper is affected by the changes at its resonant frequency and quality factor. The values of quality factor and resonant frequency of the microgripper increase when the elastic modulus of the gripped cells increment. Higher elastic modulus of the gripped cells can generate larger resonant frequencies and quality factors of the microgripper. These variations of the resonant frequency and quality factor can be used to discriminate between different cells types, determining their elasticity modulus by analyzing the frequency response curve traced with experimental results and studying the changes previously commented. The accuracy and reliability of the results will depend on the conditions of the experimental setup. Thus, the elasticity modulus values of the cells estimated through microgripper will be employed to distinguish CTCs from healthy cells.

Considering the results shown in Figure 17, the dynamic behavior of the microgripper is affected by the changes at its resonant frequency and quality factor. These variations can be used to discriminate between different cells types, determining their elasticity modulus. Thus, the elasticity modulus values of the cells estimated through microgripper will be employed to distinguish CTCs from healthy cells. For the general case, the resonant frequency is defined by:

(41) $$f_{rz}=\frac{1}{2\pi }\sqrt{\frac{k_{Tz}+\alpha_{a}}{J_{b}}}.$$

The elastic modulus of each gripped cell by the microgripper can be approximated by:

(42) $$E=\frac{2T_{cell}}{\theta_{z}r_{c}^{2}}\left(\frac{3(1-v^{2})}{4a}-\frac{f(a)}{\pi }\right),$$

with

(43) $$T_{cell}=B\sin(\omega t)-J_{b}{\ddot{\theta }}_{z}-c_{amT}{\dot{\theta }}_{z}-k_{Tz}\theta_{z}.$$

**Figure 19:**
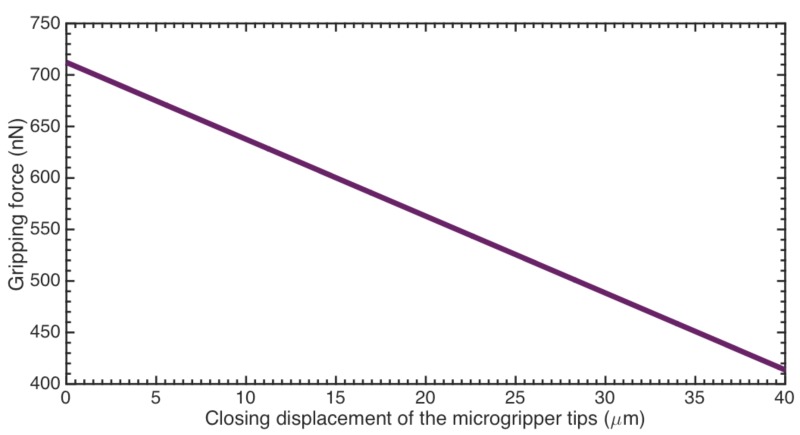
Gripping force as function compressed distance of the microgripper.

The maximum dc and ac actuation voltages are 23.4 volts and 129.2 volts, respectively. The difference between these voltage values is due to that electrodynamic comb-drive actuators have lower capacitance. Voltages *V*_0_ and *V*_1_ have the same magnitude (64.58 V) in order to maximize the actuation torque B. If the limit dc voltage is applied, the mobile arm could rotate up to 0.382 rad that allows a maximum displacement of 95.5 µm between the microgripper tips. Due to that real distance between the microgripper tips is 40 µm, the resulting energy is used to increase the gripping force. Figure 19 shows the reduction of the gripping force when the microgripper tips is closing. It is due to that mobile arm requires more energy to achieve a larger rotation angle (*θ_z_*). Thus, if the gripped cell has larger size then the gripping force magnitude must be increased. The gripped cell size affects the rotation angle of the mobile arm *θ*. If the cell has a larger size then the mobile arm will have a less rotation angle. Considering the maximum ac actuation voltage, the maximum force of the microgripper is 1.21 µN, which is enough to characterize the mechanical properties of the cell with large elastic modulus (E ≈ 9700 Pa). Based on Equation (32), the designed microgripper can measure the elastic modulus and stiffness of CTCs with diameters less than 18.34 μm. The microgripper even is capable to characterize other elastic micro-objects with bigger elastic modulus. This microgripper can be implemented at the tip of a manipulator located on anti-vibration table to improve the alignment position of the cell between fixed and mobile arm. A misalignment between the cell and the fixed and mobile arms could modify the contact area with the microgripper tips, which will affect the measurements of the stiffness and elastic modulus of the cell.

In this microgripper design, we considered that cell is characterized by the microgripper outside of the isotonic fluid environment. Thus, we only included the air-damping model. However, the microgripper could test the cell inside an isotonic fluid environment, in which we would include the fluid-damping model. This fluid damping will be larger than the air damping, which will increase the total damping force and decrease the quality factor of the microgripper. It decreases the value of the rotational resonant frequency of the microgripper.

## 4. Conclusions

A novel MEMS microgripper with rotatory electrostatic comb-drive actuators to measure the elastic modulus and stiffness of cells is presented. The designed microgripper is based on the SUMMiT V surface micromachining process from Sandia National Laboratories. This microgripper is formed by a compact structure of four polysilicon layers that operate at its rotational vibration mode in plane *xy*. This structure is composed by a mobile arm, a fixed arm and two comb-drive actuators that decrease the microgripper size, keeping a simple microgripper operation. The design of the two rotatory electrostatic comb-drive actuators allows the real-time control of both opening and closing displacements (with a range close to 40 μm) of the microgripper tips by adjusting the dc and ac bias voltages. The increment of the electrodes number without expanding the working area is achieved with the proposed design, which decreased of the size of the actuation mechanism. Analytical and FEM models were developed to determine the mechanical behavior of the microgripper with and without gripped cells. The results of the analytical models agreed very well with those obtained with the FEM simulations. This microgripper could characterize different types of biological cells with elastic modulus up to 9700 Pa. The maximum dc and ac bias voltages were 23.4 and 129.2 V, respectively. Based on the results of the mechanical characterization of the cells, the proposed microgripper could be used as a dispositive to identify CTCs from patient blood samples and study the efficiency of medical treatments against cancer.

Future research work will include the fabrication and characterization of the designed microgripper considering different dc and ac bias voltages. In addition, the microgripper will be used to determine the mechanical properties of the CTCs.

## Figures and Tables

**Table 1:** Dimensions of the electrodes for the electrodynamic actuators of the microgripper**.**

Parameter	Value
*R*_0_	400 μm
*θ*_0_	6°
*g*	2 μm
*h*	7 μm
*W_f_*	2 μm

**Table 2:** Stiffness values of the serpentine springs of the microgripper.

Stiffness	Analytical Model	FEM Model Using Beam188 Elements	Relative Difference	FEM Model Using Solid187 Elements	Relative Difference
*k_tz_*	49,557 μN μm rad^−1^	49,019 μN μm rad^−1^	−1.1%	49,901 μN μm rad^−1^	0.7%
*k_tx_*	11,982 μN μm rad^−1^	11,737 μN μm rad^−1^	−2.0%	12,280 μN μm rad^−1^	2.5%
*k_Fx_*	25.74 μN μm^−1^	25.50 μN μm^−1^	−0.9%	26.01 μN μm^−1^	1.1%
*k_Fy_*	90.76 μN μm^−1^	90.30 μN μm^−1^	−0.5%	89.45 μN μm^−1^	−1.4%
*k_Fz_*	10.14 μN μm^−1^	10.18 μN μm^−1^	0.4%	10.32 μN μm^−1^	1.8%

**Table 3:** Simulated vibration modes of the microgripper obtained by FEM.

Vibration Mode	Modal Shape	Resonant Frequency (Hz)
1	Rotational around *x*-axis	238
2	Rotational around *z*-axis	463
3	Rotational around *x*-axis	2525
4	Rotational around *y*-axis	3179
5	Rotational around *x*-axis	7352

**Table 4:** Radius and elastic modulus of different cells types [30].

Cell	Cell Type	Elastic Modulus (Pa)	Radius (μm)
BHP	Benign prostate cell	2797 ± 491	10
PC-3	Malignant prostate cell	1401 ± 162	10
LNCaP	Malignant prostate cell	287 ± 52	10

**Table 5:** Parameters to adjust the function *T_c_* for each cell type.

Cell	Parameter of the Regression (*α_a_x*^3^ + *α_b_*)	
	*α_a_*	*α_b_*
BHP	5.69 × 10^−8^	19.39 × 10^−3^
PC-3	2.85 × 10^−8^	9.69 × 10^−3^
LNCaP	5.83 × 10^−9^	19.87 × 10^−4^
